# Endovascular Interventions of Cancer-Associated Venous Thromboembolism with Symptomatic Iliocaval Venous Thrombosis: A Case Report

**DOI:** 10.3390/medicina60081369

**Published:** 2024-08-22

**Authors:** Shin Rui Ng, Jui-Chih Chang, Jin-You Jhan

**Affiliations:** 1 Division of Cardiovascular Surgery, Department of Surgery, Hualien Tzu Chi Hospital, Buddhist Tzu Chi Medical Foundation, Hualien City 970473, Taiwan; shin710132@gmail.com (S.R.N.); medraytw@hotmail.com (J.-C.C.); 2 Department of Surgery, School of Medicine, Tzu Chi University, Hualien City 97004, Taiwan

**Keywords:** venous thromboembolism, endovascular, cancer

## Abstract

Cancer-associated venous thromboembolism (CAT) poses a severe threat, disrupting ongoing cancer management and adversely impacting treatment outcomes. CAT often leads to a two- to six-fold increase in mortality rates when it progresses to venous total occlusion. The primary modalities employed in addressing this life-threatening complication include anticoagulant therapy only or coupled with strategic endovascular interventions. Aggressive endovascular interventions, such as mechanical thrombectomy and venous stent implantation, are crucial in mitigating thrombotic complications, relieving symptoms, and improving this vulnerable population’s overall quality of life and life expectancy. This case report presents a CAT case extending to the total occlusion of the inferior vena cava. Our goal is to provide valuable insights into the evolving management of CAT and its sequelae, showcasing treatment approaches that lead to improved outcomes and a better quality of life for cancer patients facing these additional challenges.

## 1. Introduction

Cancer-associated thromboembolism (CAT) poses a substantial risk to cancer patients and is the second-leading cause of mortality in this population [1,2]. Tumor secretions that alter coagulation function and tumor compression causing venous stasis significantly to elevate the risk of venous thromboembolism (VTE) [3,4]. This case report examines the intricate management of cancer-associated symptomatic iliocaval deep vein thrombosis (DVT) in a patient with advanced urothelial carcinoma, with a specific focus on aggressive invasive therapy. These interventions aim to address thrombotic complications and improve the patient’s quality of life through endovascular techniques [5].

## 2. Detailed Case Description

In this case, a 59-year-old female with type II diabetes mellitus and hypertension was diagnosed with stage IV low-grade upper tract urothelial carcinoma in the right renal pelvis, with extensive metastasis to the lymph nodes, liver, adrenal gland, lung, and bone within the past year. She had been receiving chemotherapy and immunotherapy for four months in the Department of Urology. Venous thromboembolism (VTE) with right lower limb edema had developed two months before during her second chemotherapy regimen. She was initially treated with anticoagulants (60 mg of low molecular weight heparin twice a day for the first seven days, followed by modified 15 mg of rivaroxaban due to persistent hematuria events) for cancer-associated VTE. However, the patient experienced progressive edema and swelling, extending to both lower limbs within one week after her fourth chemotherapy regimen and first immunotherapy regimen.

**Figure 1:**
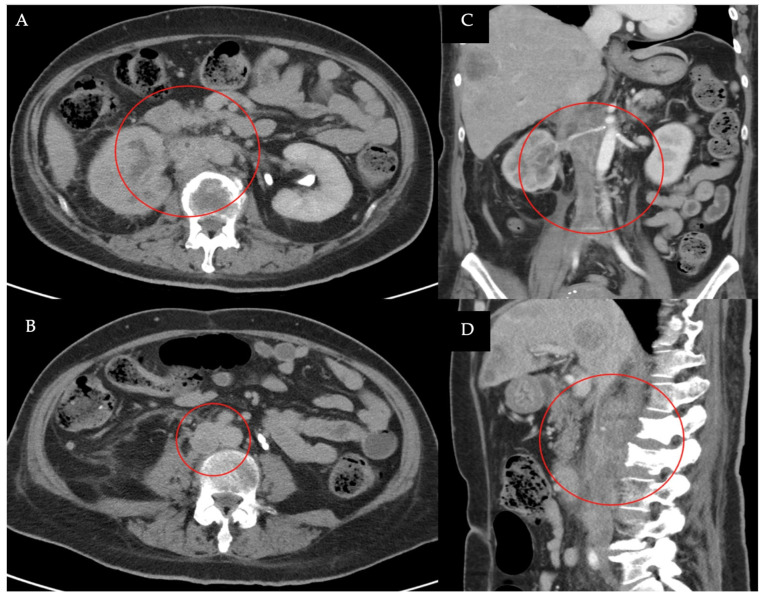
CT examination with the contrast enhanced: (**A**) Axial CT image showed heterogenous contrast-enhancing mass from right renal invaded the IVC (red circle); (**B**,**D**) Coronal CT and sagittal CT images showed heterogenous contrast-enhancing mass was noted in the IVC from CIVs to the level of renal veins (red circle); (**C**) Axial CT image showed irregular mass obstruction was noted in IVC at the level of bifurcation of CIVs (red circle).

**Figure 2:**
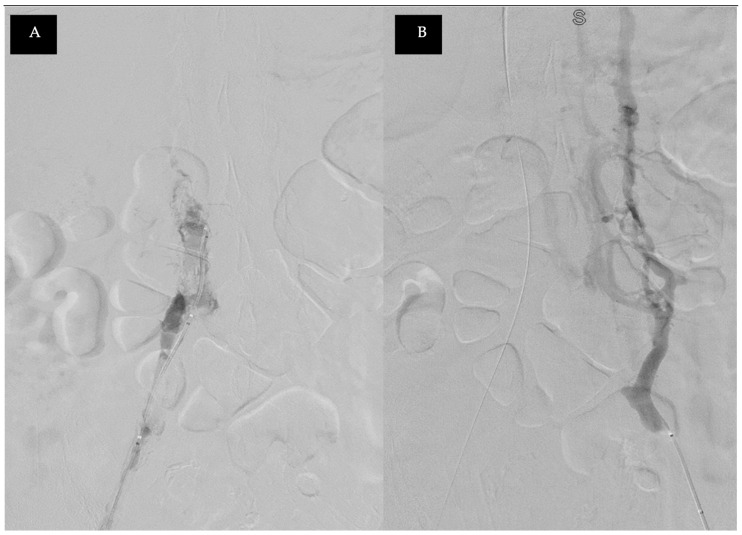
Venography: (**A**) the right common iliac vein and IVC thrombus were found; (**B**) the left common iliac vein chronic total occlusion with the patent collateral branch.

A Computed Tomography (CT) examination found right lower limb edema, revealing central venous obstruction from the bilateral common iliac veins (CIV) to the inferior vena cava (IVC) at the renal vein level (Figure 1), likely due to a combination of tumor and blood thrombus. She was referred to a vascular specialist for further evaluation. Venography showed total occlusion with tumor thrombi from the common iliac veins to the IVC (Figure 2). A series of staged endovascular interventions were planned.

**Figure 3:**
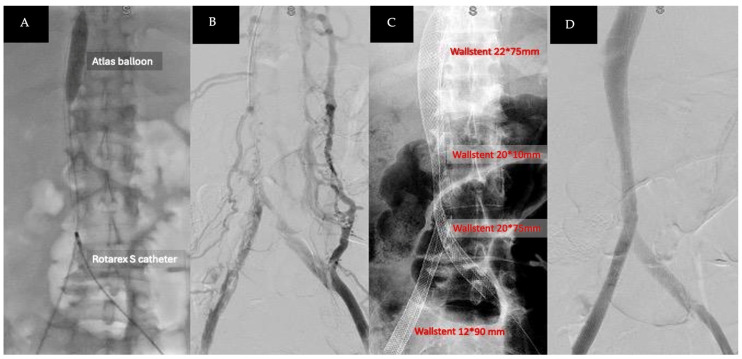
(**A**) Percutaneous mechanical thrombectomy performed with ROTAREX™ S thrombectomy catheter and PTA dilatation balloon to occlude the portal vein; (**B**) post-thrombectomy venography showed improved blood flow; (**C**) VENOUS WALLSTENT™ self-expanding stents were implanted from bilateral CIVs to IVC; (**D**) post-stent implantation venography showed patent venous flow from bilateral CIVs to IVC.

**Figure 4:**
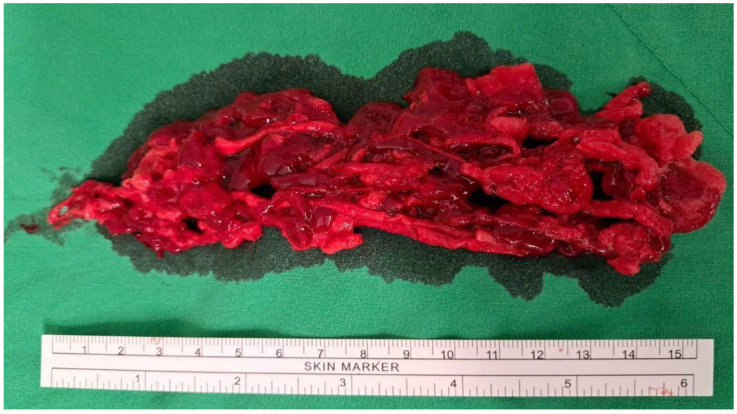
Well-organized and fresh thrombus was removed by 8-French ROTAREX™ S thrombectomy catheter.

Thrombolysis was performed using a fountain catheter inserted from the bilateral common femoral veins into the IVC. Urokinase infusion was initially administered via the fountain catheter; however, there was an abrupt decrease in fibrinogen levels, prompting the replacement with a continuous heparin infusion for anticoagulant therapy, which was maintained for 24 h. Percutaneous mechanical thrombectomy was then carried out with an 8-French ROTAREX™ S thrombectomy catheter (BD, Franklin Lakes, NJ, USA), along with the inflation of an Atlas™ Gold Percutaneous Transluminal Angioplasty (PTA) dilatation balloon catheter (BD, Franklin Lakes, NJ, USA) to occlude the portal vein entry and prevent a thrombus-induced acute pulmonary embolism (Figure 3A). A substantial amount of combined tumor and blood-organized thrombi was removed (Figure 4). Post-thrombectomy venography revealed improved contrast-enhanced blood flow from the bilateral CIVs to the IVC (Figure 3B).

An intravascular ultrasound (IVUS) device was used for lesion identification and localization and to measure the diameter of stenotic lesions from the bilateral CIVs to the IVC. VENOUS WALLSTENT™ self-expanding stents (Boston Scientific, Marlborough, MA, USA) were then deployed, matching the venous size from the portal vein to the bifurcation of the bilateral CIV and using the kissing-stent technique to extend to the external iliac vein (Figure 3C). Post-stent implantation venography showed patent venous blood flow from the bilateral CIVs to the IVC (Figure 3D).

The patient was prescribed 5 mg of apixaban (a direct oral anticoagulant, DOAC) twice daily to reduce the risk of recurrent thrombosis. Her lower limb edema significantly improved, with the regression of edema from the bilateral thighs to the feet after endovascular interventions and no recurrence of edema during hospitalization. Unfortunately, due to cancer pain and the progression of cancer-related complications, the patient received palliative care and expired one month later.

## 3. Discussion

Cancer-associated thromboembolism (CAT) presents a formidable challenge in cancer care, characterized by significant risks and high mortality rates among patients [1]. The risk of venous thromboembolism (VTE) is notably higher in individuals with cancer than in those without, across all age categories [6]. Over the last two decades, the incidence of VTE in cancer patients has increased three-fold and is nine times higher than in the general population [7]. It is estimated that up to 20 percent of patients with cancer will be affected by VTE, with the highest risk periods associated with hospitalizations and the development of metastatic disease [8]. This intricate condition can be explained through Virchow’s triad: blood stasis, hypercoagulability, and endothelial injury—factors that are equally applicable to CAT [9]. Tumor-induced alterations in coagulation increase the susceptibility to venous thromboembolism (VTE), complicating the management of cancer patients [3,4]. 

A case report illustrates the complex management of cancer-associated ileocaval venous thrombosis in a patient with advanced urothelial carcinoma, highlighting the profound impact of aggressive invasive therapies on this relentless disease [5]. These therapies demonstrate a commitment to improving patient quality of life through strategic endovascular techniques, showcasing both the challenges and successes in the evolving landscape of CAT management.

Standard guidelines typically recommend anticoagulant medication to prevent further thrombus formation in CAT. The American Society of Clinical Oncology (ASCO) suggests initial anticoagulation with LMWH, unfractionated heparin (UFH), fondaparinux, or rivaroxaban [10]. For patients initiating treatment with parenteral anticoagulation, LMWH is preferred over UFH for the initial 5 to 10 days of treatment in cancer patients with newly diagnosed VTE with no renal impairment [10,11]. For long-term anticoagulation, LMWH, edoxaban, or rivaroxaban for at least six months is preferred due to their efficacy superior to vitamin K antagonists (VKAs) [10,11]. 

However, endovascular interventions are emerging as a novel approach, offering effective palliative relief with improved tolerance and reduced invasiveness. Successful outcomes of endovascular treatments for CAT progressing to ileocecal thrombosis underscore the efficacy of these aggressive therapies. When conservative medical therapy fails or proves incompatible, particularly following strategic endovascular stent implantation, these interventions offer a viable option [5].

A significant gap exists in understanding and clinical attention regarding vena cava occlusion intertwined with tumor thrombi. This condition can extend to critical veins, such as the hepatic and renal veins, leading to visceral organ failure due to venous congestion. Recognizing these severe outcomes drives the prescription of aggressive venous mechanical thrombectomy, which plays an influential role in mitigating these progressive consequences. Mechanical thrombectomy is an endovascular technique that physically removes the thrombus without thrombolytic medications [12]. A single-center retrospective study of 90 cancer patients with deep vein thrombosis (DVT) undergoing mechanical thrombectomy showed an 87% technical success rate, with a 77% rate of intervention-free survival at six months post-procedure [12]. This strategic management aims to alleviate the burden of tumors or thrombi, providing relief for patients grappling with the complex interplay of cancer and thromboembolism [5]. 

Venous stenting, especially under intravascular ultrasound (IVUS) guidance, should be considered in cases of CAT. IVUS can detect venous lesions missed by venography and offers a more accurate assessment of stenosis [13]. IVUS was used to confirm IVC and iliofemoral occlusion, determine lesion extent, and confirm re-entry. Proximal and distal landing zones for stent placement were determined using IVUS imaging in venous segments with the least occlusive disease [13]. IVUS examination before stent deployment significantly reduces the risk of 30-day and 2-year stent reintervention compared to using multiplanar venography alone [14].

Endovascular recanalization and stent implantation offer symptomatic relief and defense against late-stage complications [15].Endovascular venous stenting is a safe procedure and should be considered as part of the palliative care for patients with debilitating lower extremity symptoms related to iliocaval and iliofemoral venous compression [16]. A retrospective study with thirty-seven cancer patients with VTE who underwent iliofemoral venography and stent implantation showed a 78 percent rate of clinical improvement in symptom relief [16]. The primary patency of the stents at 1, 3, and 6 months was 93 percent, 81 percent, and 69 percent, respectively [16]. This approach enhances quality of life amidst the challenges of CAT. The success rates of endovascular interventions are notably high, with a 95.1 percent success rate and 12-month primary patency rates reaching up to 74.1 percent [17]. With a mean follow-up of 18 months, secondary patency rates are 87 percent [18].

Regarding prophylaxis and post-intervention care, using direct oral anticoagulants (DOACs) following venous stent implantation emerges as a vital advancement. DOACs are an emerging option for acute VTE treatment, although LMWH remains an acceptable standard [2]. However, patients with active cancer are at an increased risk of bleeding complications due to the cancer itself, as well as treatments such as anticoagulants, chemotherapy, and radiotherapy [8]. The safety and efficacy of DOACs in preventing recurrent thrombus formation are comparable to standard care LMWH [3]. With a lower risk of bleeding and superior quality-of-life improvements, DOACs are essential in thromboembolism prophylaxis for this patient population [19].

In the complex landscape of cancer care, aggressive endovascular interventions represent a significant advancement in addressing the immediate complications of thromboembolism while promoting a holistic approach to patient well-being. For patients with CAT and ileocaval venous thrombosis, percutaneous thrombectomy combined with stent graft treatment, alongside pharmacological therapy, may offer an improved quality of life. Pharmaco-mechanical thrombectomy, coupled with thrombolysis using a lytic agent, is a more recent treatment modality that synergistically removes a more significant thrombus burden in a shorter treatment time with fewer complications [20]. 

## 4. Conclusions

In conclusion, this case highlights the management of cancer-associated thromboembolism (CAT) with ileocaval venous thrombosis in a patient with advanced urothelial carcinoma. Personalized treatment, including anticoagulation, mechanical thrombectomy, and stent implantation, is crucial for balancing immediate relief with long-term outcomes. Endovascular interventions play a vital role in improving the quality of life for patients with CAT.

## Data Availability

Data are contained within the article.
